# Exploration of Social Proximity and Behavior in Captive Malayan Tigers and Their Cubs

**DOI:** 10.3390/ani13061040

**Published:** 2023-03-13

**Authors:** Aimee Holland, Elena Giulia Galardi, Martina Fabbroni, Anita Hashmi, Jerome Catinaud, Richard Preziosi, James Edward Brereton, Giovanni Quintavalle Pastorino

**Affiliations:** 1 School of Science and the Engineering, Manchester Metropolitan University, Manchester M15 6BH, UK; 2 School of Biological and Marine Sciences, Plymouth University, Plymouth PL4 8AA, UK; 3 Parc Des Félins, 77540 Lumigny-Nesles-Ormeaux, France; 4 University Centre Sparsholt, Westley Lane, Sparsholt, Winchester SO21 2NF, UK; james.brereton@sparsholt.ac.uk

**Keywords:** Felidae, *Panthera tigris jacksoni*, tiger, solitary, affiliative, tolerance, sociality, welfare

## Abstract

**Simple Summary:**

With growing concern for animal welfare, understanding social behavior in captive animals is critical when making evidence-based management decisions. The sociality of large felids in captivity has remained largely understudied, yet these species are frequently housed together in practice in zoological collections. The aim of this study was to investigate the social behavior between a pair of Malayan tigers and their twin 6-month-old cubs. The aim was to investigate the welfare effects of keeping big cats in shared enclosure spaces. Using video footage, we found that the male tiger engaged in affiliative behavior, not just with his mate but also with his offspring. Even in the absence of clear affiliative interaction, the male displayed high tolerance to both conspecifics, as evidenced by his acceptance of direct physical contact and low occurrences of aggression. The findings suggest that some felid species may have greater social flexibility than has previously been documented and indicate that housing male tigers with their mates and young offspring may be a feasible management strategy in some zoological collections.

**Abstract:**

The survival of endangered felids is becoming increasingly dependent on the successful management and breeding of reserve populations in captivity. While most felid species are reported to be solitary in the wild, increasing evidence suggests that some big cats have greater social plasticity than is currently acknowledged. This social plasticity allows felids to be sometimes socially housed in environments such as zoos and rescue centers. While the effects of such shared enclosures remain in question, many reports provide evidence of several welfare benefits of maintaining these large carnivores in pairs or even groups. Since 2019, Le Parc des Félins has housed a breeding pair of Malaysian tigers (*Panthera tigris jacksoni*) alongside their offspring. The purpose of this study was to quantify the social affiliation between the male tiger and his cubs and to investigate the female’s tolerance toward him. The data were collected using video recordings in the outdoor enclosure when social interactions were observed. The data were coded and categorized in the open-source software BORIS, from which behavioral activity budgets were calculated. Data were analyzed using the chi-squared test for association to determine differences in affiliative frequency, with directed and undirected sociograms created to visualize individual relationships. Overall, the male regularly engaged in affiliative behaviors with the cubs, with no significant difference found in the frequency of interactions with them compared to the female. No physical aggression was directed by the male toward the cubs. Although the female maintained a stronger bond with the cubs compared to the male, he displayed a greater range of affiliative behaviors toward them than male tigers are thought to exhibit. Both adults showed a high degree of tolerance toward their conspecifics, suggesting that maintaining breeding pairs with their offspring is a viable management strategy in zoological collections. This study could therefore improve husbandry and conservation practices by developing our understanding of felid sociality and the potential welfare benefits of social housing, allowing for evidence-based captive management decisions.

## 1. Introduction

Sociality refers to the extent to which animals interact or engage in long-term or transient social groups [1]. For social, group-living animals, the benefits of intraspecific cooperation tend to outweigh the costs associated with such a social system [2]. It is widely documented that for many species, social relationships promote individual health, welfare, and fitness [3], so the study of sociality is essential for providing evidence-based captive management advice [4]. With growing concern for animal welfare, collecting data on social preferences can facilitate sound decision-making to structure populations and reduce social stress [5]. While most social animals have a species-specific range of affiliative behaviors, investigations into intragroup social relations within carnivores have been restricted to a few species [6], such as the spotted hyena (*Crocuta crocuta)* [7]. Although the 37 species of Felidae exemplify great diversity when it comes to size, coloration, vocalization, and habitat requirements [8], virtually all extant species are thought to be solitary in the wild [9]. While lions (*Panthera leo*) and cheetahs (*Acinonyx jubatus*) are purported to be the most socially complex of all Felidae, forming social groups in the wild [10], ecological theory notes that solitary species fare better when hunting dispersed prey in complex environments [11]. Thus, most felids are known to spend the majority of their time unaccompanied, limiting interaction to specific direct social events [8]. When combined with their elusive nature and low population densities [12], big cats are challenging to observe in the wild. This has made research on felid social structure challenging and has resulted in even scarcer studies regarding their affiliative behavior.

Indeed, previous literature suggests that females are generally intolerant of any conspecifics with the exception of their young offspring and males during mating [13]. Likewise, due to their territorial nature, males are known to be highly aggressive toward one another and only interact with females during times of courtship and copulation [14]. Offspring rearing in males is largely unheard of, with infanticide reported as the most widely documented cause of intraspecific felid mortality, namely by unrelated males [15]. Despite this, there is a growing body of literature that suggests felids are capable of exhibiting a greater repertoire of social behaviors than is currently recognized. Early work on tigers (*Panthera tigris*) documented individuals occasionally socializing and traveling in groups [16], in addition to engaging in cooperative hunting behaviors [17]. More recently, Pirie et al. [18] highlighted that male leopards (*Panthera pardus*) can maintain non-aggressive contact with their adult offspring, suggesting males are more tolerant of cubs they have sired. While males are not generally known to assist in offspring rearing, such research highlights the capacity for kin recognition in solitary felids with long-term associations between adult males and their offspring. This suggests that this research question may have merit across a wider range of felids.

The value of social strategies in elucidating rare social interactions observed in solitary animals is not yet well studied. This limits current knowledge on the cognition of less social species [19]. When investigating a group of 13 wild pumas (*Puma concolor*), Elbroch et al. [19] found a high degree of conspecific tolerance and interaction across all individuals in the network with direct reciprocity and food sharing serving as a fitness benefit and social activity for those that participated. Such research demonstrates that solitary carnivores are more socially flexible than previously thought and provides evidence that the social affiliation and tolerance observed in zoos are not just an artifact of captivity. 

While most felids are considered to be solitary in the wild, many species are not antisocial and do not necessarily need to be housed alone [20]. Thus, maintaining captive felids in pairs, trios, or even groups has become an increasingly common practice, which some scholars suggest provides several welfare benefits [21]. Compared to more social animals, solitary species may have fewer forms of enrichment as they are not typically housed with others [22]. Social housing could therefore function as a successful enrichment strategy [23,24]. Likewise, recent research on socially housed captive felids has shown that males do form social bonds with their offspring [25] and engage in parental care behaviors, with intermittent separation having adverse effects on male welfare [26]. Despite the increased use of social housing, there is limited research on its effects, with the few existing studies revealing conflicting results, so it is essential that further research be conducted on the impact on welfare and optimal grouping sizes to substantiate such practices [27].

Given that over 45% of felids are either vulnerable or endangered in the wild according to the International Union for Conservation of Nature (IUCN) Red List [28], their managed breeding in captive reserve populations is essential to their conservation. With the exception of a few independent studies, affiliative behaviors in known ‘solitary’ felids remain seldom studied or acknowledged [21,29].

It is important to identify whether Malayan tiger males (*Panthera tigris jacksoni)* can be safely housed alongside their cubs during the rearing process. This study investigates the existing literature and provides new evidence based on a case study group of tigers. This may be used to determine whether ‘solitary’ species do have greater social plasticity than is currently widely acknowledged. In doing so, it also aims to aid conservation breeding programs by informing animal husbandry practices and providing evidence of the welfare and reproduction benefits associated with social housing.

The purpose of this study was to quantify the types and frequency of affiliative behaviors displayed by a male Malayan tiger toward his cubs when socially housed at Le Parc des Félins. The objectives were to describe the frequency, nature, and scale of any present social interactions and assess the role of mothers in male–cub affiliate relations as well as her behavior toward the father. It is important to note that tolerance, even in the absence of clear affiliative behaviors, is still particularly relevant both from the father toward the cubs and from the mother toward the male in the cub’s presence. The findings in this report show that adult male tigers are capable of sustaining social bonds both with their mates and young offspring, and this may have implications for informing evidence-based management decisions in the social housing of captive tigers and other solitary felids.

## 2. Materials and Methods

### 2.1. Study Site and Animals

This study took place at Le Parc des Félins, a zoological park in Lumigny-Nesles-Ormeaux, France, dedicated to the breeding, conservation, and research of the most endangered members of the Felidae family. Situated in the Fortelle forest, with a total of 150 felids belonging to 40 species or subspecies housed over 71 hectares of land which represents one of the lowest densities of animals per hectare in Europe, the park has been able to fulfill its ethos of creating vast and naturalistic environments to promote the well-being of the animals and allow them to express their natural behaviors [30]. These conditions also permit studies such as this by allowing the observation of species in environments that more closely mimic those of their wild counterparts. 

**Figure 1:**
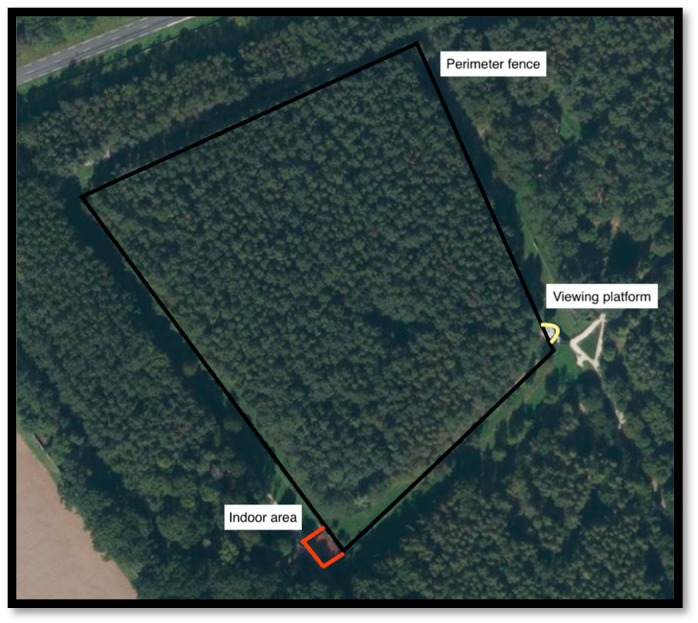
Annotated image of outdoor Malaysian tiger enclosure provided by Parc des Félins.

The animals studied were a Malayan tiger family group, consisting of a breeding pair and their twin 6-month-old female cubs (Table 1, Appendix B for images) who are socially housed at the park. The identification of individual cubs was determined by differences in coloration and facial markings. The cats have access to 2.5 hectares of forest (the world’s largest tiger enclosure) [31], providing an opportunity to observe them in a naturalistic setting and capture social interactions that are often challenging in the wild [12]. The outdoor enclosure (Figure 1) was the main focus of the study, with the tigers captured on video when they were visible from the surrounding fence line. The extent of the enclosure boundary provides multiple viewing perspectives for researchers and the public alike, including a raised visible platform that overlooks the enclosure and a train that runs along the outer perimeter of the park.

### 2.2. Observational Data

Observational data on the Malayan tigers were obtained between the 22nd and 30th of January 2020. The tigers were monitored using video recordings taken by keepers over two sessions each day (morning and afternoon). Focal animal behavioral observations [32] were utilized as the most satisfactory method of studying groups as they produce accurate data on the frequencies and durations of behaviors of interest [33]. Similarly, all-occurrence and ad libitum sampling techniques [32] were employed to create an exact record of state behaviors as well as to capture any relevant events [34]. While the latter method may suffer from bias toward those behavior patterns and individuals which tend to be most conspicuous, it is particularly useful for capturing those rare but important interactions that this study seeks to understand [34].

An ethogram was constructed specifically to analyze father–cub interactions after sample viewing of the collected videos. By tailoring it to the tigers in this report we were able to describe and quantify subtle, unique interactions. Generic behaviors were based on a standardized felid version developed by Stanton et al. [35] and previous research conducted by Quintavalle Pastorino et al. [36,37]. Behaviors were adapted to fit those displayed within the project, with social behaviors indicating the direction to identify who initiated and received an interaction. In order to create activity budgets for each individual, all behaviors were recorded as state behaviors, and behaviors were categorized into classes by assessing analogous behavioral descriptions and the purpose of displayed behaviors (Table 2). Likewise, a review of current literature allowed for the comparison of common behaviors and thus the refining of definitions and categorization.

### 2.3. Data Coding

The ethogram and the recordings taken by the keepers were uploaded to the open-source software BORIS allowing for user-specific, coding-based observations to be conducted of the selected subjects [38]. Each individual was entered as a separate subject, with an additional generic cub subject used when a cub was unidentifiable. A total of 339 videos were used for data analysis and each video was coded individually for each tiger that was present. While time-consuming, the advantage of coding data in this way is that it provides an accurate method of timing behavior and allows the record to be analyzed repeatedly and in different ways [34].

### 2.4. Data Analysis

Data analysis was conducted using a combination of the built-in analysis tools in BORIS Desktop (v.7.9.2, University of Torino, Italy, 2019), Microsoft Excel (version 16.48, Microsoft, Manchester, United Kingdom, 2021), and R Studio (version 1.2.5033, R Studio Inc., 2019). Time budgets were produced in BORIS for each subject relating to behavioral categories and individual behavior occurrences. The data from the BORIS outputs were exported to Microsoft Excel and organized to formulate relevant tables that would be used for further analysis. R Studio was used to conduct statistical tests to assess differences in the frequency and scale of affiliative interactions between individuals. Moreover, a sociogram was constructed using the igraph package within R Studio to examine the relationships within the family unit, particularly between the male and his cubs. A sociogram is a form of social network diagram [35,36] that demonstrates the strength of affiliative associations between individuals [39]. Individual and global network metrics were calculated to quantify and describe the nature of relationships, such as the strength and density of associations and the connectedness of individuals. As the cubs were identifiable, they were able to be entered as individual nodes. The generic cub subject was removed from the analysis as there was only one occurrence of affiliative interaction where the cub could not be distinguished.

## 3. Results

### 3.1. Activity Budgets

#### 3.1.1. Frequency of Behavioral Categories

**Figure 2:**
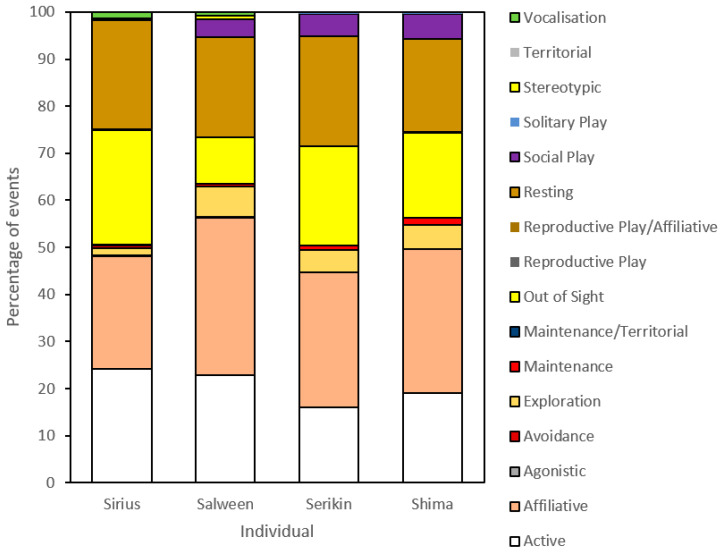
Activity budget of overall frequency (%) of behavioral observations for the male (Sirius), female (Salween), and two cub tigers (Serikin and Shima) at Parc des Félins.

Figure 2 displays the overall frequency of behavioral observations for the male, female, and cub tigers. Overall, all four tigers exhibited similar frequencies in each behavioral category, with each displaying ‘affiliative’ as their highest behavioral frequency. Salween and Shima each displayed a slightly higher frequency of this behavior than Sirius, with a maximum 4.07% difference between him and Salween. Serikin, on the other hand, displayed a marginally lower frequency than Sirius, with a 0.84% difference. The second highest category for all tigers was ‘active’, with Sirius (21.14%) and Salween (23.57%) showing similar frequencies. However, the majority of Sirius’s events consisted of patrolling (11.16%) while in Salween’s case it was walking (12.05%). While affiliative interactions were similar between the four tigers, ‘social play’ was substantially greater for Salween and the two cubs in comparison to Sirius. ‘Reproductive play’, while low for Sirius and Salween, was non-existent amongst the cubs, which is unsurprising given such events are indicative of adult mating behaviors. There were few agonistic behaviors displayed by all individuals, with only 1.79% for Sirius and 1.05% for Salween. The cubs showed no initiated agonistic behaviors, with one received by Shima. Likewise, ‘avoidance’ was low in all tigers, with Sirius displaying no behaviors in this category. Sirius was observed ‘vocalizing’ more often than both Salween and the cubs, as would be expected from a territorial male.

#### 3.1.2. Duration of Behavioral Categories

**Figure 3:**
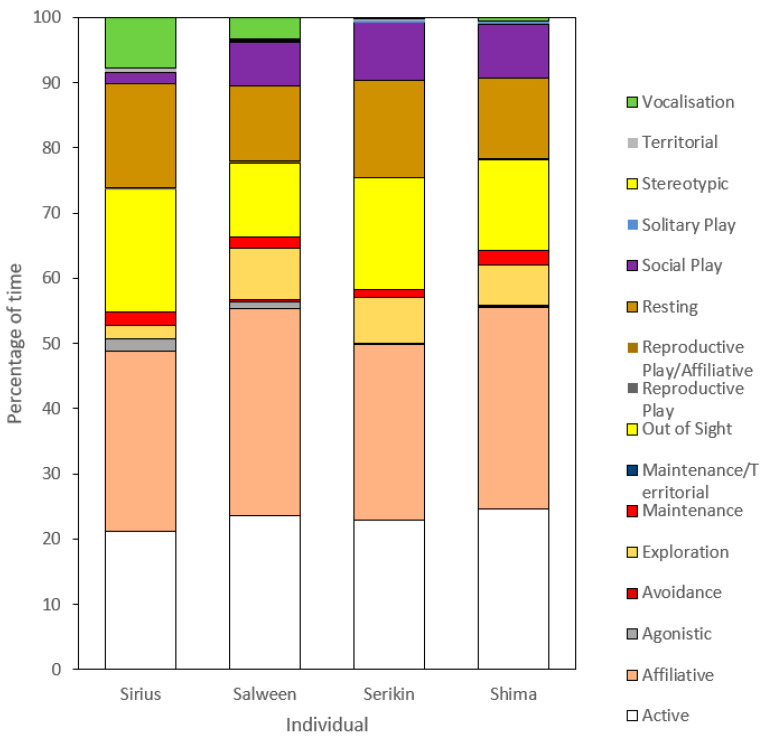
Activity budget of overall duration (%) of behavioral observations for the male (Sirius), female (Salween), and two cub tigers (Serikin and Shima) at Parc des Félins.

Figure 3 displays the overall duration for each behavioral category for the male, female, and cub tigers. Again, there are several similarities in some behavioral budgets between the four tigers; however, there are also substantial differences compared to those seen in behavioral frequency. While ‘affiliative’ remains the highest category for Shima and the cubs, Sirius’s largest duration is ‘out of sight’, closely followed by ‘active’. The difference in affiliative duration is more pronounced than with frequency, with a 9.65% difference between Sirius and Salween and an average of 5.68% between Sirius and the cubs. Similar results are reported for social play, with Salween and, in particular, the cubs displaying higher durations than Sirius, whose duration for this category is less than 1%. ‘Agonistic’ behaviors were rarely seen and amounted to less than 1% of the budget in all four tigers, with Sirius displaying the highest at 0.23%. Similarly, ‘avoidance’ was not observed in Sirius and Serikin and occurred infrequently in Salween (0.05%) and Shima (0.03%). Sirius again ‘vocalizes’ for a longer duration than the other tigers; however, the difference is much less explicit than with frequency. The most substantial difference found was Salween being ‘out of sight’ on average 21.23% less than Sirius and the cubs, indicating she was observed more often in the recordings. This is supported by her having both the highest number of behavioral events and the longest durations.

#### 3.1.3. Affiliative vs. Agonistic Behaviors

**Figure 4:**
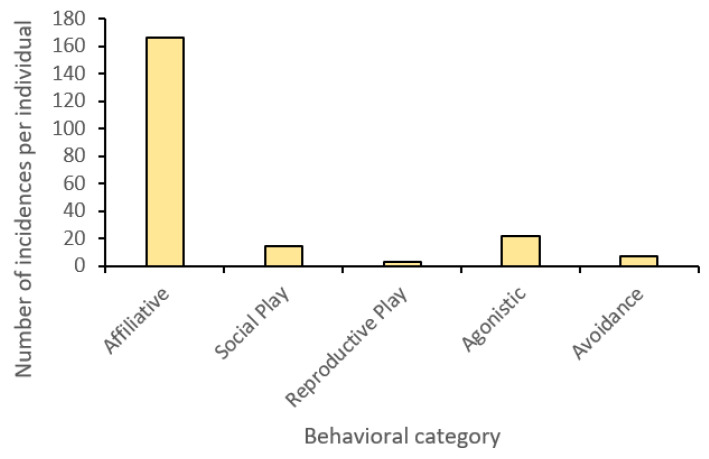
Behavioral category frequency for social interactions between the male (Sirius) and female (Salween) tigers (undirected) at Parc des Félins.

Sirius–Salween: Throughout the entirety of the research period, aggressive (‘agonistic’) behaviors directed from the male to the female occurred infrequently, with most cases transpiring in vocal aggression (e.g., roar) as opposed to physical aggression (e.g., fight, warning bite). Instances that did result in physical aggression were exclusive to feeding times. Salween only initiated one act of aggression toward Sirius, with a comparatively greater tendency to demonstrate avoidance. ‘Affiliative’ behaviors were observed to occur seven times more frequently than aggressive (Figure 4), with chuffing, head rubbing, and staring as some of the most common behaviors. ‘Reproductive play’ was less common with Salween initiating all interactions. ‘Social play’ was also minimal compared to affiliative interactions and limited to ‘pawing’ and ‘head butting’, which were primarily directed toward Sirius by Salween. Sirius was less likely to initiate social interactions with his mate, yet he was highly receptive to those directed toward him, highlighting a level of tolerance between him and Salween.

**Figure 5:**
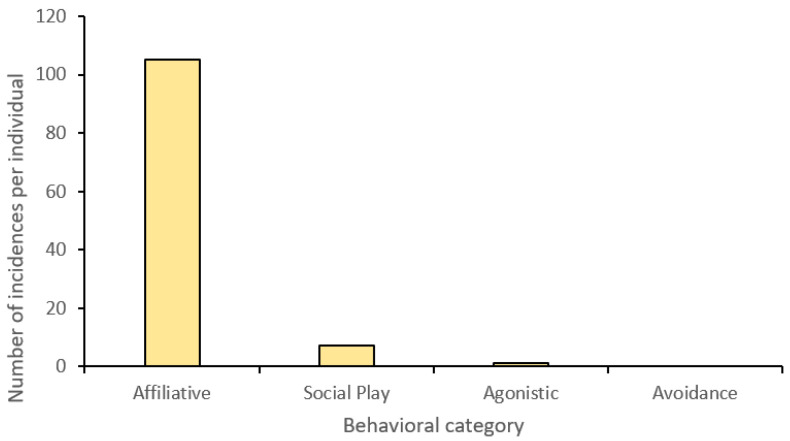
Behavioral category frequency for social interactions between the male (Sirius) tiger and the two cubs (Serikin and Shima) (undirected) at Parc des Félins.

Sirius–cubs: No instances of physical aggression directed by the male toward either of his cubs were observed (see Figure 5). Aggressive behaviors were absent, with the exception of a single ‘Roar’ directed at Shima. Likewise, there were no instances of avoidance from Sirius with the cubs, suggesting he was tolerant of their presence around him. While Sirius displayed a narrower range of affiliative behaviors in comparison to his mate, he still exhibited a wider repertoire than would be expected from an adult male, with occurrences of ‘chuffing’, ‘heading rubbing’, and ‘sniffing’ present. Moreover, he spent a considerable amount of his resting time in proximity to his cubs, although he was never in direct contact, resting near or within a body length of one or both of them, further supporting his acceptance of them. Similarly, in his relationship with Salween, Sirius initiated fewer interactions between himself and the cubs, yet he was tolerant of those received and would reciprocate occasionally.

### 3.2. Social Network Analysis 

#### 3.2.1. Sociograms

**Figure 6:**
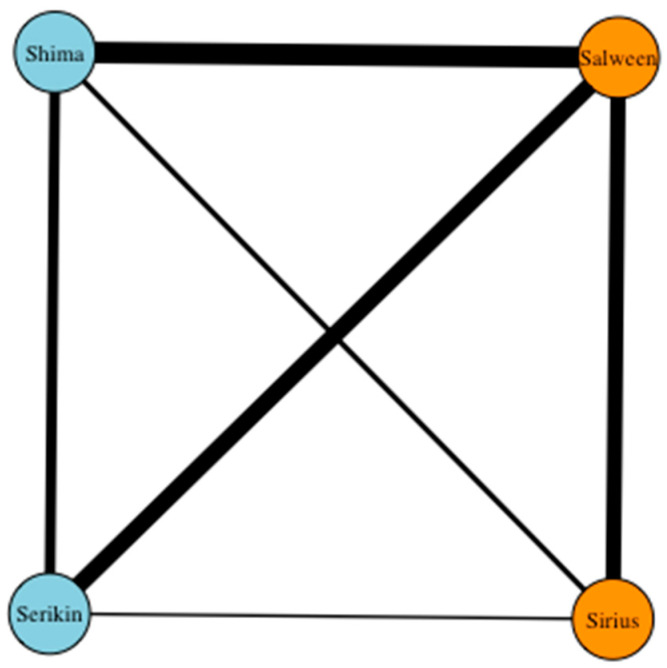
Sociogram displaying the total frequency of affiliative interactions (undirected) between the male, female, and cub tigers at Parc des Félins classed by age (orange = adult, light blue = cub). Edges are weighed based on total number of interactions between two nodes.

The sociogram in Figure 6 highlights the frequency of undirected affiliative interactions between the male, female, and cub tigers. The strongest relationship shown is between Salween and the cubs, particularly Shima, and it was to be expected that the female would be the primary caregiver. Sirius, while displaying a weaker affiliation with the cubs by comparison, still demonstrates a connection with both, although again slightly stronger with Shima, suggesting that she may be the more social or outgoing cub. Even in the absence of clear affiliative interaction, Sirius displays a high degree of tolerance toward the cubs. Salween and Sirius also share a strong association, which is not only indicative of a reciprocated bond but also the high level of tolerance they display toward one another. For Salween, this is particularly significant as she is highly tolerant of Sirius’s presence around the cubs, an atypical response from an adult female.

**Figure 7:**
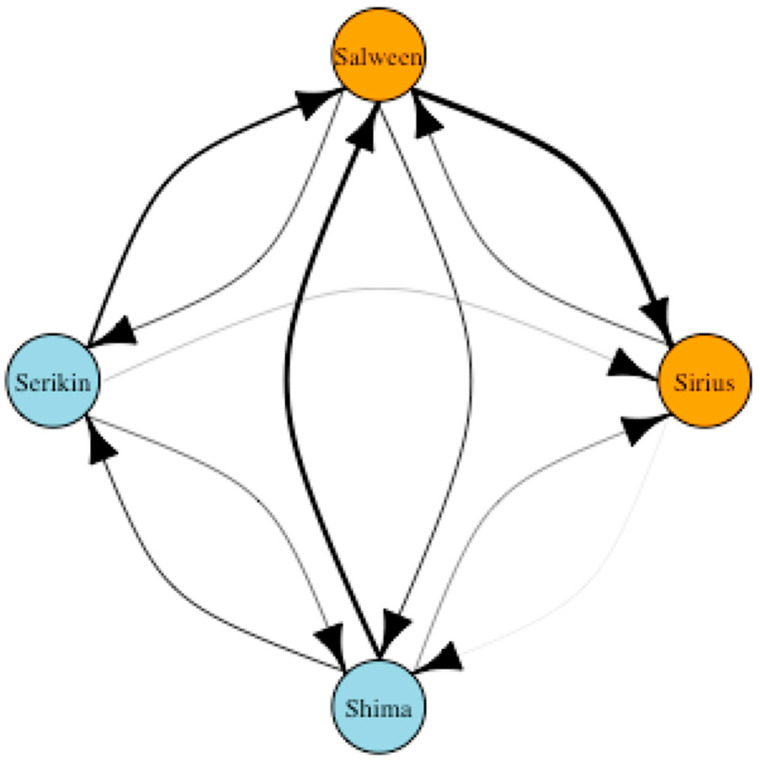
Sociogram displaying the frequency of affiliative interactions (directed) between the male, female, and cub tigers at Parc des Félins classed by age (orange = adult, light blue = cub). Edges are weighed based on total number of initiated interactions between two nodes.

The sociogram in Figure 7 highlights the frequency of directed affiliative interactions between the male, female, and cub tigers. Similar to the undirected network, Salween has a strong relationship with both of the cubs; however, the directed network highlights that the cubs initiate a higher frequency of interactions compared to Salween, who directs more of her interactions at Sirius. While the cubs share an affiliation, they both initiate more interactions with Salween than they do with each other. Sirius has a connection to both of the cubs; however, it is evident that his relationship with Shima is stronger as he receives more interactions from her and does not direct any interactions at Serikin. The greatest number of interactions initiated is from Salween to Sirius, further emphasizing the levels of tolerance she demonstrates toward her mate. Sirius directs the majority of his interactions toward Salween, indicating they do share a reciprocated affiliation, albeit unevenly weighted.

#### 3.2.2. Network Metrics

Analysis of the observed patterns identified within the social network can be quantified by calculating global network metrics (Table 3). Network density is high at 1 suggesting the family has high levels of cohesiveness, with all members associating with one another. This is supported by both the low mean path length and diameter (1) indicating all individuals are well connected. However, it is important to take into account group size; as Faust (2006) notes, it can be assumed density must decrease with population size, hence this level of connectedness is likely due to the relatively small sample size. Moreover, the global clustering (GC) is also high, suggesting that the family is more clustered around certain dyads rather than equally across the network.

Individual network metrics can be calculated to describe an individual’s position in the network and their relative association patterns (Table 4). Analysis of node degree highlights that all individuals are well connected within the network, with the maximum number of direct ties. From node strength, it is evident Salween interacts more frequently than the other tigers and maintains the strongest connections. This is further supported by her high level of betweenness in comparison to the others, indicating she may act as a central link between individuals in the network. While Sirius associates less frequently than Salween and the cubs, he still exhibits a comparable number of interactions, a rare finding for an adult male tiger.

### 3.3. Statistical Tests

A chi-square association test was conducted to test the affiliative interactions between the dyads. The results showed there was no significant difference in affiliative interaction between Salween and the cubs compared to Sirius and the cubs (X-squared = 2.5692, df = 1, and *p*-value = 0.109) suggesting they all associate similarly. Such a finding was supported by testing for differences in the total number of interactions between all individuals. A Kruskal–Wallis test was used as the data were not normally distributed. Likewise, there was no significant difference found (chi-squared= 3, df = 3, and *p*-value = 0.3916), implying any variance seen in interactions between the tigers is not substantial.

## 4. Discussion

With increasing concern for animal welfare, an evidence base is needed when making management decisions such as keeping animals in social groups [4]. For those wishing to breed endangered species in zoos, collecting information concerning individual social preferences ensures appropriate housing choices whilst minimizing social stress [5]. Despite their solitary nature, many wild felids have been observed participating in social groups, yet their affiliative behaviors remain understudied [29]. As wild observations continue to be challenging, social housing in captivity provides a unique opportunity to examine an animal’s social behavior on a long-term basis which is often not possible in situ [40]. Overall, this case study shows that male tigers are capable of forming social bonds, not only with their mates but also with their own offspring. Even in the absence of clear affiliative interaction, the male displayed high tolerance to both conspecifics, as evidenced by his acceptance of directed contact and low occurrences of aggression. While the evidence found is promising in revealing father–cub affiliative behavior, it is important to note the small sample size involved, with further research needed to substantiate such claims. Furthermore, when interpreting the data, it is essential to consider that other factors such as personality, enclosure design and size, husbandry procedures, period of social housing, and reduced competition from captive conditions may play a role in the social plasticity observed in the tigers [36].

Individual activity budgets showed that all four tigers exhibited affiliation as their highest behavioral category. These findings were to be expected as recordings were taken when tigers were seen associating. However, it is interesting to note that all individuals displayed a similar number of affiliative events. Males are not believed to interact with their offspring in the wild [15], yet this finding shows that they possess a greater capacity for non-aggressive interaction than is currently widely documented. There were few agonistic and avoidance behaviors exhibited by all individuals, suggesting that there was a high degree of tolerance between all conspecifics. While not conducted in this study, it may have been prudent to conduct personality assessments in order to determine whether specific personality types are associated with successful group-housing [35,36]. Social play was higher among the female and the cubs, which was to be expected as mothers and juveniles have been commonly reported to engage in play [41]. The male was observed to vocalize more often than either the female or the cubs and often exhibited such behavior when external stimuli were present, such as visitor noise and roaring from other felids in the park. In terms of roaring, such communication is thought to warn other conspecifics of an individual’s presence and location [41]. As vocalizations were frequently accompanied by patrolling, it is likely that these behaviors have emerged from normal guarding of the territory [42], with neighboring cats acting as ‘competitors’ [43].

Time budgets for overall behavioral duration revealed that the adult male spent more time ‘resting’ and less time ‘active’ when compared to the female. Like most felids, tigers are known to spend a significant amount of the day inactive, with males typically more active than females [44]. While the findings of this study contradict such a notion, they mirror those found by Quintavalle Pastorino et al. [45] which suggest the male was more inactive as a result of the female’s vigilance and care of the cubs. However, the male did spend considerably more of his time (12.7%) patrolling than the female which is typical of a male controlling his territory [46]. In captivity, an animal walking different routes within the enclosure may emulate their need to perform locomotive behaviors and patrol their home range [21]. Moreover, while males are not known to partake in offspring rearing [18], patrolling may also act as a form of cub guarding, in which the female allowed the male to protect the cubs, thus sharing, if only in part, parental care.

When examining affiliative relationships, the male maintained the strongest association with his mate, directing most of his interactions toward her, with minimal instances of physical aggression observed. Anecdotally, the rare occasion in which such behavior did occur was limited to times of feeding. As wild adult tigers are not generally thought to engage in food sharing in the wild [41], it is unsurprising that the male acted territorially during feeding. However, a previous study has documented male tigers allowing females to feed from their kill [41], indicating they do have the capacity for such social behavior. Affiliative behavior was more frequently observed than agonistic, with chuffing, head rubbing, and staring as some of the most common behaviors. While the female was more likely to initiate any observed social interactions, the male was highly receptive to those directed toward him and would respond at times. It is clear that not only do adult tigers share a strong association, often characterized by reciprocity, but there is also a high degree of tolerance displayed toward one another. While this is significant for the males as they are the less social sex and are highly aggressive to other conspecifics due to their territorial nature [14], this is arguably more telling of the females. Although females do associate more often than males in times of mating and offspring rearing [47] they are highly intolerable of other conspecifics, particularly males when they have dependent young due to the risk of infanticide [15]. Females have been known to kill males in defense of cubs [48]. The natural instinct to be protective around their young may be a biologically driven strategy [15]. Yet, the female only initiated one act of aggression toward the male and not in the presence of the cubs. The fact that she allowed the male to spend time in the proximity of the cubs and actively chose to engage in affiliative interaction with him suggests a level of trust which has enabled them to cohabit efficiently. Likewise, this may have also been developed as a strategy to coax or mollify the male, reinforcing the notion that the female was highly tolerant of his presence around both her and the cubs. It is reasonable that their amicable and often affiliative relationship is a result of their shared history; the two have been cohabiting since they were first introduced in 2016 and have had two previous litters. Captive management of socially housed felids would therefore benefit from further research to determine if the period of cohabitation and the number of litters together impacts relationships in terms of tolerance and affiliative bonds. In doing so, we may be able to ascertain a baseline age for cubs to be introduced to their father with minimal risk, whilst also reducing any unnecessary stress for the female in relation to the father’s presence. Similarly, it would be valuable to establish if enclosure size has any effect, as the uniquely large outdoor area allowed the tigers to avoid other conspecifics if desired, which may have helped alleviate any social stress.

No instances of physical aggression directed from the male to either of his cubs were observed and only one occurrence of vocal aggression. In the wild, some male felids (mainly unrelated) regularly practice infanticide in order to both reduce competition and the amount of time before the female becomes receptive again [49]. Yet, the lack of agonistic or avoidance behaviors suggests the male was tolerant of the cubs’ presence, further signifying the social flexibility that has been previously underestimated in solitary male felids. As expected, the female, as the primary caregiver, shares the strongest relationship with the cubs compared to the male. However, the male does still demonstrate a connection to both, exhibiting a wider repertoire of behaviors than would be anticipated from an adult male, with occurrences of ‘chuffing’, ‘heading rubbing’, and ‘sniffing’ present. Both relationships are stronger toward one cub (Shima), suggesting she may be the more social or outgoing cub of the two. Again, the male was less forthcoming in initiating interactions with the cubs; however, he was amenable to those received and would occasionally reciprocate. Even in the absence of clear social interaction, the male exhibits a high level of tolerance toward the cubs, reaffirming his acceptance of them. This is further evidenced by the considerable amount of his resting time spent in the proximity of one or both of the cubs, yet never being in direct contact. Given that the tigers have access to over 2.5 hectares of outdoor space, it is surprising that the male tiger was observed in a large proportion of the recordings with either the cubs or the female, suggesting he chose to spend time in the proximity of one of them. This emphasizes the notion that the male was accepting of his conspecific’s presence. Similar evidence of affiliative bonds between relatives has been found in other tiger studies. When tigers were maintained with a relative such as a brother, sister, or half-sister, De Rouck et al. [21] revealed that affiliative interactions were more likely to be exhibited between littermates. As Hunter et al. [50] suggest, while males are not thought to engage in parental caregiving behaviors, they are more tolerant of offspring they have sired. Indeed, Pirie et al. [18] found evidence of longer-term associations between an adult male and two generations of his offspring. They observed an adult male leopard maintaining non-aggressive contact with his adult offspring, including friendly greeting behaviors such as heard rubbing and tail wrapping. This tolerance exemplifies the capacity for kin recognition in solitary felids that has previously not been documented. The findings from this report suggest that social plasticity between groups may be more common within felids than has previously been acknowledged. Similar to the relationships found by Quintavalle Pastorino et al. [45], the affiliation and tolerance observed between the father and his cubs offer a promising solution for alternative management strategies when housing felids in captivity.

As solitary animals often fare worse in captivity compared to more social ones due to a lack of conspecific interaction [22], the social housing of more solitary species has been considered a successful enrichment strategy [23]. While such practices have become increasingly common in zoological collections, there remains a lack of research into the effects of cohabitation on well-being [21], with existing studies revealing conflicting results [27]. Macri and Patterson Kane [51] observed 18 captive snow leopards aged between 11 months and 16 years and found that while solitary cats were more active with greater displays of pacing, social cats engaged in a wider range of species-specific behaviors, including direct social interactions and vocalizations. As snow leopards are known to be capable of forming pair bonds, denying sexually mature individuals access to mates in captivity may be a form of stress, particularly in their breeding years [52]. Hence, understanding the effect of age on cohabitation calls for further study. Conversely, an investigation into a group of six two-year-old female tigers by Miller and Kuhar [29] over a six-year period revealed that non-contact aggression and vocalization increased while social proximity decreased. Excluding a few independent studies, there is little information concerning the feasibility of housing felids in large social groups and none on same-sex groupings [27]. While the literature reveals conflicting results on the effects of social housing and optimal age/sex groupings, this study provides a potentially viable social model of a reproductive couple and cubs up to 6 months of age for socially housing conspecific tigers. It can be suggested that such a system may benefit the welfare of individuals by allowing them to fulfill their social needs and establish strong social and affiliative bonds. This is further evidenced by the minimal aggressive, avoidance, or stereotypic behaviors observed, suggesting the animals were not suffering from social stress. Although the results of this study are by no means conclusive, the findings are promising, with further studies on the impact on welfare and optimal grouping size, age, and sex ratio warranted to substantiate the practice of social housing that is already in place in some institutions [27].

While social behaviors in solitary felids are more commonly observed in zoos, there is evidence to show that this is not just an artifact of captivity. For example, research has found that males do associate occasionally with females and their offspring, spending time in their presence and even showing displays of affection in terms of licking cubs and sharing kills [48]. Likewise, in 2015, camera traps used as part of the Wildlife Conservation Society’s Russia Program captured for the first time an adult male accompanying a female Amur tiger and cubs [53]. Not only does this demonstrate that males do partake in family life, if only occasionally [53], but it also reminds us how little we really know about the sociality of wild felids. Indeed, our understanding of the cognition of less social species has been hindered due to previous research ignoring the value of social strategies in explaining rare social interactions in solitary species [19]. Analysis of 13 wild pumas revealed a network comprising densely connected communities in which all individuals participated. Conspecific tolerance was high, with food sharing serving as a social activity and fitness benefit for those involved [19]. The findings illustrate that solitary felids have greater social plasticity than is currently understood which allows them to adapt to the present environmental and ecological conditions [54], a strategy previously reserved for group-living species [55]. Captive felids are not subject to the same environmental pressures such as the availability of resources that determine optimal group size in wild populations. As such factors are controlled for, the animals experience little to no competition compared to their wild counterparts, meaning that group size is more flexible in captivity [23]. It is conceivable that the animals in this study were able to cohabit amiably and adapt to a social system characterized by a high degree of tolerance and direct affiliative interaction due to the lack of ecological pressures and conspecific competition. What is clear is that our current knowledge of the social ability of solitary felids is evidently flawed; therefore, future research should examine the selective and ecological pressures animals are subjected to, to better predict the emergence of complex social strategies. Successful management of felids in captivity requires a better understanding of an individual’s social capacity and needs that may differ from the minimal degree of sociality many wild felids have been observed to conform to.

## 5. Conclusions

Despite a growing body of literature indicating otherwise, current research has overlooked the significance of rare social interactions in ‘solitary’ felids resulting in inadequate descriptions of their societies [56]. As Macdonald et al. [15] note, an animal’s solitary nature does not exclude it from a complex social life. Nevertheless, with the exception of a few independent cases, the study of affiliative interactions has been seldom considered in typically ‘solitary’ felids. Indeed, the results of this study reveal that some males can form social bonds with both their mates and with their own offspring. The high level of tolerance demonstrated by both the female toward the male and the male toward his cubs, even in the absence of clear affiliative interaction, is an indication that housing male felids with their mates and young offspring may be a feasible management strategy in some zoological collections. Indeed, these data highlight the potential for other institutions when considering social housing, although it is important to take into account the age and sex of individuals. Such plasticity may not have been observed within a different dynamic such as if the cubs were of a sexually mature age. Thus, while our research may document a successful experience of keeping a male tiger with his young offspring, the findings should not be generalized beyond this study but should instead serve as a reference model in the context of other institutions’ collections. Likewise, although not a focus of this study, individual character and temperament may play a significant role in the plasticity of the parents, so personality assessments may be critical for informing compatibility when socially housing felids [35,36]. It is therefore recommended that future research focus on other typically solitary felids, with multiple assessments of welfare to be conducted on both adults and cubs in order to substantiate the practice of social housing that is already in place in some collections [45]. While the evidence found in this study may also be attributed to factors such as enclosure design, husbandry practices, and the lack of competition for food, territory, or mates that captivity provides, it can be suggested that species such as the Malayan tiger are capable of group living, potentially in the wild, if only in certain circumstances such as favorable ecological and environmental conditions. By understanding the selective pressures individuals are subjected to, we can better predict the emergence of complex social strategies. With declining wild felid populations, understanding a species’ adaptive potential is essential for informing effective captive management and designing conservation breeding programs. Therefore, the literature would benefit from a long-term study of socially housed Felidae groups to further develop our understanding of sociality and comprehend the socio-ecological dynamics that produce rare but revealing interactions.

## Figures and Tables

**Table 1:** Individual basic information on critically endangered (IUCN 2022) Malaysian tigers housed within Parc des Félins at the time of the study.

Name	Sex	D.O.B.	Age	Relationship
Sirius	Male	02/10/2007	12	Father
Salween	Female	10/08/2011	8	Mother
Shima	Female	08/07/2019	6 months	Cub
Serikin	Female	08/07/2019	6 months	Cub

**Table 2:** Behavioral classes categorized in order to create time budgets. Individual behaviors selected come from the selected ethogram in Appendix A.

Class	Behaviors Included
Active	Walking, running, patrolling, climbing, rolling, rearing up, standing, stretching, head/body shake, tail movement, kneading
Resting	Lie, sit, crouch, belly up, eyes closed, head down, yawn
Affiliative	Approach conspecific, allogroom conspecific, body/head rub conspecific, chuff, follow conspecific, stare at conspecific, sniff conspecific, touch noses with conspecific, lick conspecific
Exploration	Flehmen, look around, sniff, touch object, chew object, dig, explore
Territorial	Body/head rub object, spray, scratch with paws, defecate
Social Play	Bite conspecific, chase conspecific, head-butt conspecific, play-fight conspecific, paw conspecific, trip conspecific, stalk conspecific, jump on conspecific
Solitary Play	Play with water/object, play roll, carry object
Reproductive Play	Nape bite, mount, sniff anogenital region
Agonistic	Warning bite, chase conspecific aggressively, fight conspecific, roar/hiss at conspecific, slap conspecific, bare teeth at conspecific
Avoidance	Avoid conspecific
Vocalization	Syndetic call, chuff, roar, hiss, grunt/cough, growl
Maintenance	Defecate
Out of Sight	Beyond one’s range of vision
Stereotypic	Pace

**Table 3:** Global network metrics from the undirected network for the male (Sirius), female (Salween), and two cub tigers (Serikin and Shima) at Parc des Félins.

Metric	Value
Density	1
Diameter	1
Global Clustering	1
Mean Path	1

**Table 4:** Individual network metrics from the undirected network for the male (Sirius), female (Salween), and two cub tigers (Serikin and Shima) at Parc des Félins.

Metric	Sirius	Salween	Serikin	Shims
Degree	3	3	3	3
Strength	324	739	421	514
Betweenness	0	2	0	0

## Data Availability

Data are available from the corresponding author upon reasonable request.

## Appendix A

Full ethogram, categorized with descriptions of behaviors and vocalizations.

**Behavior**	**Category**	**Description**
Body shake	Active	Cat rotates its abdomen from side to side
Climbing	Active	Cat ascends or descends an object or structure
Head shake	Active	Repetitive move of the head with short and quick movements
Patrolling	Active	Cat is alert and walks around in a calm, deliberate manner, periodically stopping to perform investigative or marking behaviors
Running	Active	Forward locomotion at a quick gait
Roll	Active	Lying on the ground, the animal rotates its body from side to side; during the roll, the back is rubbed against ground, the belly is exposed, and all paws are in the air
Rear up	Active	Cat stands up on its hind legs with forelegs toward or against object
Stretching	Active	Cat extends body and forelegs forward and curves the back and tail upwards
Standing	Active	Cat stands in an upright position with all four legs extended and paws on the ground, immobile
Tail twitch	Active	A rapid flick of the tail in either a side to side or up and down motion
Tail tip	Active	Prolonged, repeated movement of tip of the tail
Tail slash	Active	Standing or moving with tail bent over body, slashing
Walking	Active	Forward locomotion at a slow gait
Kneading	Active	Cat pushes forepaws into the ground or (modifier) in a rhythmic, kneading motion
Approached by Sirius and reciprocates	Affiliative	Cat reciprocates interaction with conspecific
Approached by Sirius	Affiliative	Cat is approached by conspecific
Approached by Shima and reciprocates	Affiliative	Cat reciprocates interaction with conspecific
Approached by Shima	Affiliative	Cat is approached by conspecific
Approached by Serikin and reciprocates	Affiliative	Cat reciprocates interaction with conspecific
Approached by Serikin	Affiliative	Cat is approached by conspecific
Approached by Salween and reciprocates	Affiliative	Cat reciprocates interaction with conspecific
Approached by Salween	Affiliative	Cat is approached by conspecific
Approached by cubs and reciprocates	Affiliative	Cat reciprocates interaction with conspecific
Approached by cubs	Affiliative	Cat is approached by conspecific
Approach Sirius	Affiliative	Cat moves toward conspecific while looking at it
Approach Shima	Affiliative	Cat moves toward conspecific while looking at it
Approach Serikin	Affiliative	Cat moves toward conspecific while looking at it
Approach Salween	Affiliative	Cat moves toward conspecific while looking at it
Approach observer	Affiliative	Cat initiates and seeks proximity to observer; readily approaches the fence in an amicable manner (e.g., chuffs, rubs on fence)
Approach cubs	Affiliative	Cat moves toward conspecific while looking at it
Allogroomed by Sirius and reciprocates	Affiliative	Active receiver of allogrooming from conspecific
Allogroomed by Sirius	Affiliative	Has the fur licked by a conspecific
Allogroomed by Shima and reciprocates	Affiliative	Active receiver of allogrooming from conspecific
Allogroomed by Shima	Affiliative	Has the fur licked by a conspecific
Allogroomed by Serikin and reciprocates	Affiliative	Active receiver of allogrooming from conspecific
Allogroomed by Serikin	Affiliative	Has the fur licked by a conspecific
Allogroomed by Salween and reciprocates	Affiliative	Active receiver of allogrooming from conspecific
Allogroomed by Salween	Affiliative	Has the fur licked by a conspecific
Allogroomed by cubs and reciprocates	Affiliative	Active receiver of allogrooming from conspecific
Allogroom Sirius	Affiliative	Licks the fur of a conspecific
Allogroom Shima	Affiliative	Licks the fur of a conspecific
Allogroom Serikin	Affiliative	Licks the fur of a conspecific
Allogroom Salween	Affiliative	Licks the fur of a conspecific
Allogroom cubs	Affiliative	Licks the fur of a conspecific
Body rubbed by Sirius and reciprocates	Affiliative	Active receiver of body rubbing from conspecific
Body rubbed by Sirius	Affiliative	Rubbed by a conspecific
Body rubbed by Shima and reciprocates	Affiliative	Active receiver of body rubbing from conspecific
Body rubbed by Shima	Affiliative	Rubbed by a conspecific
Body rubbed by Serikin and reciprocates	Affiliative	Active receiver of body rubbing from conspecific
Body rubbed by Serikin	Affiliative	Rubbed by a conspecific
Body rubbed by Salween and reciprocates	Affiliative	Active receiver of body rubbing from conspecific
Body rubbed by Salween	Affiliative	Rubbed by a conspecific
Body rubbed by cubs and reciprocates	Affiliative	Active receiver of body rubbing from conspecific
Body rubbed by cubs	Affiliative	Rubbed by a conspecific
Body rub Sirius	Affiliative	Rubs body on conspecific
Body rub Shima	Affiliative	Rubs body on conspecific
Body rub Serikin	Affiliative	Rubs body on conspecific
Body rub Salween	Affiliative	Rubs body on conspecific
Body rub cubs	Affiliative	Rubs body on conspecific
Chuffed by Sirius and reciprocates	Affiliative	Cat reciprocates interaction with conspecific
Chuffed by Serikin and reciprocates	Affiliative	Cat reciprocates interaction with conspecific
Chuffed by Shima and reciprocates	Affiliative	Cat reciprocates interaction with conspecific
Chuffed by Salween and reciprocates	Affiliative	Cat reciprocates interaction with conspecific
Chuffed by cubs and reciprocates	Affiliative	Cat reciprocates interaction with conspecific
Chuffed by Sirius	Affiliative	Cat receives chuff from conspecific
Chuffed by Shima	Affiliative	Cat receives chuff from conspecific
Chuffed by Serikin	Affiliative	Cat receives chuff from conspecific
Chuffed by Salween	Affiliative	Cat receives chuff from conspecific
Chuffed by cubs	Affiliative	Cat receives chuff from conspecific
Chuff at Sirius	Affiliative	Cat expels jets of air through the nose creating a low intensity, soft, pulsed sound, described as being similar to the snorting of a horse
Chuff at Shima	Affiliative	Cat expels jets of air through the nose creating a low intensity, soft, pulsed sound, described as being similar to the snorting of a horse
Chuff at Serikin	Affiliative	Cat expels jets of air through the nose creating a low intensity, soft, pulsed sound, described as being similar to the snorting of a horse
Chuff at Salween	Affiliative	Cat expels jets of air through the nose creating a low intensity, soft, pulsed sound, described as being similar to the snorting of a horse
Chuff at cubs	Affiliative	Cat expels jets of air through the nose creating a low intensity, soft, pulsed sound, described as being similar to the snorting of a horse
Chuff at observer	Affiliative	Cat expels jets of air through the nose creating a low-intensity, soft, pulsed sound; directed at observer as a friendly greeting
Followed by Sirius	Affiliative	Conspecific walks behind
Followed by Shima	Affiliative	Conspecific walks behind
Followed by Serikin	Affiliative	Conspecific walks behind
Followed by Salween	Affiliative	Conspecific walks behind
Followed by cubs	Affiliative	Conspecific walks behind
Follow Sirius	Affiliative	Walk behind conspecific
Follow Shima	Affiliative	Walk behind conspecific
Follow Serikin	Affiliative	Walk behind conspecific
Follow Salween	Affiliative	Walk behind conspecific
Follow cubs	Affiliative	Walk behind conspecific
Facing observer	Affiliative	Cat initiates and seeks proximity to observer; readily approaches the fence in an amicable manner (e.g., chuffs, rubs on fence)
Head rubbed by Sirius	Affiliative	Head rubbed by conspecific
Head rubbed by Shima	Affiliative	Head rubbed by conspecific
Head rubbed by Serikin	Affiliative	Head rubbed by conspecific
Head rubbed by Salween	Affiliative	Head rubbed by conspecific
Head rubbed by cubs	Affiliative	Head rubbed by conspecific
Head rubbed by Sirius and reciprocates	Affiliative	Active receiver of head rubbing from conspecific
Head rub Sirius	Affiliative	Rubs head on conspecific
Head rubbed by Shima and reciprocates	Affiliative	Active receiver of head rubbing from conspecific
Head rub Shima	Affiliative	Rubs head on conspecific
Head rubbed by Serikin and reciprocates	Affiliative	Active receiver of head rubbing from conspecific
Head rub Serikin	Affiliative	Rubs head on conspecific
Head rubbed by Salween and reciprocates	Affiliative	Active receiver of head rubbing from conspecific
Head rub Salween	Affiliative	Rubs head on conspecific
Head rub mesh (observer)	Affiliative	Rubs head on mesh near observer
Head rub cubs and reciprocates	Affiliative	Rubs head on conspecific
Head rub cubs	Affiliative	Rubs head on conspecific
Rest near Sirius	Affiliative	Within one body length of another animal
Rest near Shima	Affiliative	Within one body length of another animal
Rest near Serikin	Affiliative	Within one body length of another animal
Rest near Salween	Affiliative	Within one body length of another animal
Rest near cubs	Affiliative	Within one body length of another animal
Rest body contact with Sirius	Affiliative	In body contact with conspecific
Rest body contact with Shima	Affiliative	In body contact with conspecific
Rest body contact with Serikin	Affiliative	In body contact with conspecific
Rest body contact with Salween	Affiliative	In body contact with conspecific
Rest body contact with cubs	Affiliative	In body contact with conspecific
Stared at by Sirius and reciprocates	Affiliative	Cat reciprocates interaction with conspecific
Stared at by Sirius	Affiliative	Stared at by conspecific
Stared at by Shima and reciprocates	Affiliative	Cat reciprocates interaction with conspecific
Stared at by Shima	Affiliative	Stared at by conspecific
Stared at by Serikin and reciprocates	Affiliative	Cat reciprocates interaction with conspecific
Stared at by Serikin	Affiliative	Stared at by conspecific
Stared at by Salween and reciprocates	Affiliative	Cat reciprocates interaction with conspecific
Stared at by Salween	Affiliative	Stared at by conspecific
Stared at by cubs and reciprocates	Affiliative	Cat reciprocates interaction with conspecific
Stared at by cubs	Affiliative	Stared at by conspecific
Stare at Sirius	Affiliative	Looks fixedly at someone
Stare at Shima	Affiliative	Looks fixedly at someone
Stare at Serikin	Affiliative	Looks fixedly at someone
Stare at Salween	Affiliative	Looks fixedly at someone
Stare at cubs	Affiliative	Looks fixedly at someone
Sniffed by Sirius and reciprocates	Affiliative	Cat reciprocates interaction with conspecific
Sniffed by Sirius	Affiliative	Cat is sniffed by conspecific
Sniffed by Shima and reciprocates	Affiliative	Cat reciprocates interaction with conspecific
Sniffed by Shima	Affiliative	Cat is sniffed by conspecific
Sniffed by Serikin and reciprocates	Affiliative	Cat reciprocates interaction with conspecific
Sniffed by Serikin	Affiliative	Cat is sniffed by conspecific
Sniffed by Salween and reciprocates	Affiliative	Cat reciprocates interaction with conspecific
Sniffed by Salween	Affiliative	Cat is sniffed by conspecific
Sniffed by cubs and reciprocates	Affiliative	Cat reciprocates interaction with conspecific
Sniffed by cubs	Affiliative	Cat is sniffed by conspecific
Sniff Sirius	Affiliative	Cat smells the anogenital region of another cat
Sniff Shima	Affiliative	Cat smells the anogenital region of another cat
Sniff Serikin	Affiliative	Cat smells the anogenital region of another cat
Sniff Salween	Affiliative	Cat smells the anogenital region of another cat
Sniff cubs	Affiliative	Cat smells the anogenital region of another cat
Tail up	Affiliative	Tail is held vertically, in an upright position
Vocalization—Mew	Affiliative	The distinctive meow call that is typical of cats
Touch noses with Sirius	Affiliative	Two cats sniff at and touch each other with their noses
Touch noses with Salween	Affiliative	Two cats sniff at and touch each other with their noses
Touch noses with Serikin	Affiliative	Two cats sniff at and touch each other with their noses
Touch noses with Shima	Affiliative	Two cats sniff at and touch each other with their noses
Touch noses with cubs	Affiliative	Two cats sniff at and touch each other with their noses
Nurse Shima	Affiliative	Cat nurses cub or cub attempts to nurse from mother
Nurse Serikin	Affiliative	Cat nurses cub or cub attempts to nurse from mother
Warning bite at Sirius	Agonistic	Snap teeth in response to an unwelcomed closing individual
Warning bite at Shima	Agonistic	Snap teeth in response to an unwelcomed closing individual
Warning bite at Serikin	Agonistic	Snap teeth in response to an unwelcomed closing individual
Warning bite at Salween	Agonistic	Snap teeth in response to an unwelcomed closing individual
Warning bite at cubs	Agonistic	Snap teeth in response to an unwelcomed closing individual
Receives warning bite from Sirius	Agonistic	Receives warning bite from conspecific
Receives warning bite from Shima	Agonistic	Receives warning bite from conspecific
Receives warning bite from Serikin	Agonistic	Receives warning bite from conspecific
Receives warning bite from Salween	Agonistic	Receives warning bite from conspecific
Receives warning bite from cubs	Agonistic	Receives warning bite from conspecific
Belly up defense	Agonistic	Animal lies on its back with bared teeth, all four paws up with claws unsheathed
Chased aggressively by Sirius and reciprocates	Agonistic	Cat reciprocates interaction with conspecific
Chased aggressively by Sirius	Agonistic	Pursued by conspecific
Chased aggressively by Shima and reciprocates	Agonistic	Cat reciprocates interaction with conspecific
Chased aggressively by Shima	Agonistic	Pursued by conspecific
Chased aggressively by Serikin and reciprocates	Agonistic	Cat reciprocates interaction with conspecific
Chased aggressively by Serikin	Agonistic	Pursued by conspecific
Chased aggressively by Salween and reciprocates	Agonistic	Cat reciprocates interaction with conspecific
Chased aggressively by Salween	Agonistic	Pursued by conspecific
Chased aggressively by cubs and reciprocates	Agonistic	Cat reciprocates interaction with conspecific
Chased aggressively by cubs	Agonistic	Pursued by conspecific
Chase Sirius aggressively	Agonistic	Runs after conspecific
Chase Shima aggressively	Agonistic	Runs after conspecific
Chase Serikin aggressively	Agonistic	Runs after conspecific
Chase Salween aggressively	Agonistic	Runs after conspecific
Chase cubs aggressively	Agonistic	Runs after conspecific
Suffer a fight started by Sirius	Agonistic	Passive receiver of conspecific fight
Suffer a fight started by Shima	Agonistic	Passive receiver of conspecific fight
Suffer a fight started by Serikin	Agonistic	Passive receiver of conspecific fight
Suffer a fight started by Salween	Agonistic	Passive receiver of conspecific fight
Suffer a fight started by cubs	Agonistic	Passive receiver of conspecific fight
Start a fight with Sirius	Agonistic	Initiates interaction with conspecific in a harmfulmanner
Start a fight with Shima	Agonistic	Initiates interaction with conspecific in a harmfulmanner
Start a fight with Serikin	Agonistic	Initiates interaction with conspecific in a harmfulmanner
Start a fight with Salween	Agonistic	Initiates interaction with conspecific in a harmfulmanner
Start a fight with cubs	Agonistic	Initiates interaction with conspecific in a harmfulmanner
Retreat from a fight with Sirius	Agonistic	Flees from interaction started by conspecific
Retreat from a fight with Shima	Agonistic	Flees from interaction started by conspecific
Retreat from a fight with Serikin	Agonistic	Flees from interaction started by conspecific
Retreat from a fight with Salween	Agonistic	Flees from interaction started by conspecific
Retreat from a fight with cubs	Agonistic	Flees from interaction started by conspecific
Receive a fight started by Sirius and reciprocate	Agonistic	Cat reciprocates interaction with conspecific
Receive a fight started by Shima and reciprocate	Agonistic	Cat reciprocates interaction with conspecific
Receive a fight started by Serikin and reciprocate	Agonistic	Cat reciprocates interaction with conspecific
Receive a fight started by Salween and reciprocate	Agonistic	Cat reciprocates interaction with conspecific
Receive a fight started by cubs and reciprocate	Agonistic	Cat reciprocates interaction with conspecific
Rear up fight with Sirius	Agonistic	Cat stands up on its hind legs with forelegs toward or against conspecific and engages in physical combat
Rear up fight with Shima	Agonistic	Cat stands up on its hind legs with forelegs toward or against conspecific and engages in physical combat
Rear up fight with Serikin	Agonistic	Cat stands up on its hind legs with forelegs toward or against conspecific and engages in physical combat
Rear up fight with Salween	Agonistic	Cat stands up on its hind legs with forelegs toward or against conspecific and engages in physical combat
Rear up fight with cubs	Agonistic	Cat stands up on its hind legs with forelegs toward or against conspecific and engages in physical combat
Hiss at Sirius	Agonistic	A drawn-out, low-intensity hissing sound produced by rapid expulsion of air from the cat’s mouth, usually during exhalation
Hiss at Shima	Agonistic	A drawn-out, low-intensity hissing sound produced by rapid expulsion of air from the cat’s mouth, usually during exhalation
Hiss at Serikin	Agonistic	A drawn-out, low-intensity hissing sound produced by rapid expulsion of air from the cat’s mouth, usually during exhalation
Hiss at Salween	Agonistic	A drawn-out, low-intensity hissing sound produced by rapid expulsion of air from the cat’s mouth, usually during exhalation
Hiss at cubs	Agonistic	A drawn-out, low-intensity hissing sound produced by rapid expulsion of air from the cat’s mouth, usually during exhalation
Jumped on by Sirius aggressively and reciprocates	Agonistic	Cat reciprocates interaction with conspecific
Jumped on by Sirius aggressively	Agonistic	Attacked suddenly and forcefully jumped on by conspecific
Jumped on by Shima aggressively and reciprocates	Agonistic	Cat reciprocates interaction with conspecific
Jumped on by Shima aggressively	Agonistic	Attacked suddenly and forcefully jumped on by conspecific
Jumped on by Serikin aggressively and reciprocates	Agonistic	Cat reciprocates interaction with conspecific
Jumped on by Serikin aggressively	Agonistic	Attacked suddenly and forcefully jumped on by conspecific
Jumped on by Salween aggressively and reciprocates	Agonistic	Cat reciprocates interaction with conspecific
Jumped on by Salween aggressively	Agonistic	Attacked suddenly and forcefully jumped on by conspecific
Jumped on by cubs aggressively and reciprocates	Agonistic	Cat reciprocates interaction with conspecific
Jumped on by cubs aggressively	Agonistic	Attacked suddenly and forcefully jumped on by conspecific
Jump on Sirius aggressively	Agonistic	Attack suddenly and forcefully jump on the back of conspecific
Jump on Shima aggressively	Agonistic	Attack suddenly and forcefully jump on the back of conspecific
Jump on Serikin aggressively	Agonistic	Attack suddenly and forcefully jump on the back of conspecific
Jump on Salween aggressively	Agonistic	Attack suddenly and forcefully jump on the back of conspecific
Jump on cubs aggressively	Agonistic	Attack suddenly and forcefully jump on the back of conspecific
Roar at Sirius	Agonistic	Long, throaty, high-intensity call
Roar at Shima	Agonistic	Long, throaty, high-intensity call
Roar at Serikin	Agonistic	Long, throaty, high-intensity call
Roar at Salween	Agonistic	Long, throaty, high-intensity call
Roar at cubs	Agonistic	Long, throaty, high-intensity call
Receive a roar from Sirius and reciprocate	Agonistic	Cat reciprocates interaction with conspecific
Receive a roar from Sirius	Agonistic	Receives a roar from conspecific
Receive a roar from Shima and reciprocate	Agonistic	Cat reciprocates interaction with conspecific
Receive a roar from Shima	Agonistic	Receives a roar from conspecific
Receive a roar from Serikin and reciprocate	Agonistic	Cat reciprocates interaction with conspecific
Receive a roar from Serikin	Agonistic	Receives a roar from conspecific
Receive a roar from Salween and reciprocate	Agonistic	Cat reciprocates interaction with conspecific
Receive a roar from Salween	Agonistic	Receives a roar from conspecific
Receive a roar from cubs and reciprocate	Agonistic	Cat reciprocates interaction with conspecific
Receive a roar from cubs	Agonistic	Receives a roar from conspecific
Slapped by Sirius aggressively and reciprocates	Agonistic	Cat reciprocates interaction with conspecific
Slapped by Sirius aggressively	Agonistic	Cat struck by conspecific
Slapped by Shima aggressively and reciprocates	Agonistic	Cat reciprocates interaction with conspecific
Slapped by Shima aggressively	Agonistic	Cat struck by conspecific
Slapped by Serikin aggressively and reciprocates	Agonistic	Cat reciprocates interaction with conspecific
Slapped by Serikin aggressively	Agonistic	Cat struck by conspecific
Slapped by Salween aggressively and reciprocates	Agonistic	Cat reciprocates interaction with conspecific
Slapped by Salween aggressively	Agonistic	Cat struck by conspecific
Slapped by cubs aggressively and reciprocates	Agonistic	Cat reciprocates interaction with conspecific
Slapped by cubs aggressively	Agonistic	Cat struck by conspecific
Slap Sirius aggressively	Agonistic	Cat strikes conspecific
Slap Shima aggressively	Agonistic	Cat strikes conspecific
Slap Serikin aggressively	Agonistic	Cat strikes conspecific
Slap Salween aggressively	Agonistic	Cat strikes conspecific
Slap cubs aggressively	Agonistic	Cat strikes conspecific
Received bare teeth by Sirius and reciprocated	Agonistic	Cat reciprocates interaction with conspecific
Received bare teeth by Sirius	Agonistic	Is on the receiving end of bared teeth
Received bare teeth by Shima and reciprocated	Agonistic	Cat reciprocates interaction with conspecific
Received bare teeth by Shima	Agonistic	Is on the receiving end of bared teeth
Received bare teeth by Serikin and reciprocated	Agonistic	Cat reciprocates interaction with conspecific
Received bare teeth by Serikin	Agonistic	Is on the receiving end of bared teeth
Received bare teeth by Salween and reciprocated	Agonistic	Cat reciprocates interaction with conspecific
Received bare teeth by Salween	Agonistic	Is on the receiving end of bared teeth
Received bare teeth by cubs and reciprocated	Agonistic	Cat reciprocates interaction with conspecific
Received bare teeth by cubs	Agonistic	Is on the receiving end of bared teeth
Bare teeth at Sirius	Agonistic	Animal opens its mouth and pulls the lips back, exposing its teeth
Bare teeth at Shima	Agonistic	Animal opens its mouth and pulls the lips back, exposing its teeth
Bare teeth at Serikin	Agonistic	Animal opens its mouth and pulls the lips back, exposing its teeth
Bare teeth at Salween	Agonistic	Animal opens its mouth and pulls the lips back, exposing its teeth
Bare teeth at cubs	Agonistic	Animal opens its mouth and pulls the lips back, exposing its teeth
Hissed at by Shima and reciprocates	Agonistic	Cat reciprocates interaction with conspecific
Hissed at by Shima	Agonistic	Receives a hiss from conspecific
Hissed at by Serikin and reciprocates	Agonistic	Cat reciprocates interaction with conspecific
Hissed at by Serikin	Agonistic	Receives a hiss from conspecific
Hissed at by Salween and reciprocates	Agonistic	Cat reciprocates interaction with conspecific
Hissed at by Salween	Agonistic	Receives a hiss from conspecific
Hissed at by cubs and reciprocates	Agonistic	Cat reciprocates interaction with conspecific
Hissed at by cubs	Agonistic	Receives a hiss from conspecific
Avoid Sirius	Avoidance	Cat moves, or changes direction while moving, in order to keep away from conspecific
Avoid Shima	Avoidance	Cat moves, or changes direction while moving, in order to keep away from conspecific
Avoid Serikin	Avoidance	Cat moves, or changes direction while moving, in order to keep away from conspecific
Avoid Salween	Avoidance	Cat moves, or changes direction while moving, in order to keep away from conspecific
Avoid cubs	Avoidance	Cat moves, or changes direction while moving, in order to keep away from conspecific
Flehmen	Exploration	Sniffs, then lifts head with open mouth, breathes in, eyes almost closed and upper lip curled
Look around	Exploration	Turns one’s eyes toward something or in some direction in order to see
Sniffing object	Exploration	Smells object by inhaling air through the nose
Sniffing air	Exploration	Smells by inhaling air through the nose
Touch object with paw	Exploration	Cat touches object with paw
Touch object with nose	Exploration	Cat touches object with nose
Digging	Exploration	Cat breaks up or moves substrate around with its paws
Sniffing ground	Exploration	Smells by inhaling air through the nose
Chew on object	Exploration	Cat grinds an object in its mouth using the teeth
Explore	Exploration	Cat moves around attentively while sniffing the ground and/or objects
Drink	Maintenance	Cat ingests water (or other liquids) by lapping up with the tongue
Eat	Maintenance	Cat ingests food (or other edible substances) by means of chewing with the teeth and swallowing
Self-groom	Maintenance	Cat licks own fur
Lick lips	Maintenance	Protrudes tongue from the mount and lick lips
Urinate	Maintenance	Cat releases urine on the ground while in a squatting position
Retching	Maintenance	Cat makes gastric and esophageal movements of vomiting without expulsion of vomit
Nurse	Maintenance	Cub suckles or attempts to suckle from mother
Defecate	Maintenance/Territorial	Cat releases feces on the ground while in a squatting position
Out of sight	Out of Sight	Out of range of the observer’s vision
Hissed at by Sirius and reciprocates	Reproductive Play	Cat reciprocates interaction with conspecific
Hissed at by Sirius	Reproductive Play	Receives a hiss from conspecific
Mounted by Sirius and reciprocates	Reproductive Play	Cat reciprocates interaction with conspecific
Mounted by Sirius	Reproductive Play	Cat is straddled by conspecific
Mounted by Shima and reciprocates	Reproductive Play	Cat reciprocates interaction with conspecific
Mounted by Shima	Reproductive Play	Cat is straddled by conspecific
Mounted by Serikin and reciprocates	Reproductive Play	Cat reciprocates interaction with conspecific
Mounted by Serikin	Reproductive Play	Cat is straddled by conspecific
Mounted by Salween and reciprocates	Reproductive Play	Cat reciprocates interaction with conspecific
Mounted by Salween	Reproductive Play	Cat is straddled by conspecific
Mounted by cubs and reciprocates	Reproductive Play	Cat reciprocates interaction with conspecific
Mounted by cubs	Reproductive Play	Cat is straddled by conspecific
Mount Sirius	Reproductive Play	Cat attempts intromission by straddling conspecific with front and hind feet
Mount Shima	Reproductive Play	Cat attempts intromission by straddling conspecific with front and hind feet
Mount Serikin	Reproductive Play	Cat attempts intromission by straddling conspecific with front and hind feet
Mount Salween	Reproductive Play	Cat attempts intromission by straddling conspecific with front and hind feet
Mount cubs	Reproductive Play	Cat attempts intromission by straddling conspecific with front and hind feet
Nape bitten by Sirius and reciprocates	Reproductive Play	Cat reciprocates interaction with conspecific
Nape bitten by Sirius	Reproductive Play	Cat receives inhibited nape bite
Nape bitten by Shima and reciprocates	Reproductive Play	Cat reciprocates interaction with conspecific
Nape bitten by Shima	Reproductive Play	Cat receives inhibited nape bite
Nape bitten by Serikin and reciprocates	Reproductive Play	Cat reciprocates interaction with conspecific
Nape bitten by Serikin	Reproductive Play	Cat receives inhibited nape bite
Nape bitten by Salween and reciprocates	Reproductive Play	Cat reciprocates interaction with conspecific
Nape bitten by Salween	Reproductive Play	Cat receives inhibited nape bite
Nape bitten by cubs and reciprocates	Reproductive Play	Cat reciprocates interaction with conspecific
Nape bitten by cubs	Reproductive Play	Cat receives inhibited nape bite
Licked by Sirius and reciprocates	Reproductive Play/Affiliative	Cat reciprocates interaction with conspecific
Licked by Sirius	Reproductive Play/Affiliative	Cat receives lick from conspecific
Licked by Shima and reciprocates	Reproductive Play/Affiliative	Cat reciprocates interaction with conspecific
Licked by Shima	Reproductive Play/Affiliative	Cat receives lick from conspecific
Licked by Serikin and reciprocates	Reproductive Play/Affiliative	Cat reciprocates interaction with conspecific
Licked by Serikin	Reproductive Play/Affiliative	Cat receives lick from conspecific
Licked by Salween and reciprocates	Reproductive Play/Affiliative	Cat reciprocates interaction with conspecific
Licked by Salween	Reproductive Play/Affiliative	Cat receives lick from conspecific
Licked by cubs and reciprocates	Reproductive Play/Affiliative	Cat reciprocates interaction with conspecific
Licked by cubs	Reproductive Play/Affiliative	Cat receives lick from conspecific
Lick Sirius	Reproductive Play/Affiliative	Cat’s tongue protrudes from mouth and strokes conspecific
Lick Shima	Reproductive Play/Affiliative	Cat’s tongue protrudes from mouth and strokes conspecific
Lick Serikin	Reproductive Play/Affiliative	Cat’s tongue protrudes from mouth and strokes conspecific
Lick Salween	Reproductive Play/Affiliative	Cat’s tongue protrudes from mouth and strokes conspecific
Lick cubs	Reproductive Play/Affiliative	Cat’s tongue protrudes from mouth and strokes conspecific
Belly up	Resting	Animal lies on its back with throat and belly exposed to the opponent
Crouching	Resting	Cat is alert and positions the body close to the ground, whereby all four legs are bent, and the belly is touching (or raised slightly off) the ground
Decubitus—dorsal	Resting	Cat lays down on the dorsum
Eyes closed	Resting	Eyes are closed
Ears backwards	Resting	Ears orientate backward
Head down	Resting	Head is down close to ground
Decubitus—lateral	Resting	Cat lies down laterally, legs may be raised
Decubitus—sternal, lunula	Resting	Cat lies down on the sternum, legs lie to one side
Resting under wood platform	Resting	Cat lies down under platform, may or may not be sleeping
Resting near mesh	Resting	Cat lies down near mesh, may or may not be sleeping
Resting in the bushes	Resting	Cat lies down in the bushes, may or may not be sleeping
Sitting	Resting	Cat is in an upright position with all four feet on the ground, hind legs are folded, while front legs are straight and extended
Decubitus—sternal, sphynx	Resting	Cat lies down on the sternum, back legs parallel and orientated forward
Yawn	Resting	The mouth is opened widely, the head tips back, lips are pulled back so that the teeth are exposed
Bitten by Sirius and reciprocates	Social Play	Cat reciprocates interaction with conspecific
Bitten by Sirius	Social Play	Cat is bitten by conspecific in a playful way
Bitten by Shima and reciprocates	Social Play	Cat reciprocates interaction with conspecific
Bitten by Shima	Social Play	Cat is bitten by conspecific in a playful way
Bitten by Serikin and reciprocates	Social Play	Cat reciprocates interaction with conspecific
Bitten by Serikin	Social Play	Cat is bitten by conspecific in a playful way
Bitten by Salween and reciprocates	Social Play	Cat reciprocates interaction with conspecific
Bitten by Salween	Social Play	Cat is bitten by conspecific in a playful way
Bitten by cubs and reciprocates	Social Play	Cat reciprocates interaction with conspecific
Bitten by cubs	Social Play	Cat is bitten by conspecific in a playful way
Bite Sirius	Social Play	Cat snaps teeth at conspecific in a playful way
Bite Shima	Social Play	Cat snaps teeth at conspecific in a playful way
Bite Serikin	Social Play	Cat snaps teeth at conspecific in a playful way
Bite Salween	Social Play	Cat snaps teeth at conspecific in a playful way
Bite cubs	Social Play	Cat snaps teeth at conspecific in a playful way
Chased by Sirius and reciprocates	Social Play	Cat reciprocates interaction with conspecific
Chased by Sirius	Social Play	Pursued by conspecific
Chased by Shima and reciprocates	Social Play	Cat reciprocates interaction with conspecific
Chased by Shima	Social Play	Pursued by conspecific
Chased by Serikin and reciprocates	Social Play	Cat reciprocates interaction with conspecific
Chased by Serikin	Social Play	Pursued by conspecific
Chased by Salween and reciprocates	Social Play	Cat reciprocates interaction with conspecific
Chased by Salween	Social Play	Pursued by conspecific
Chased by cubs and reciprocates	Social Play	Cat reciprocates interaction with conspecific
Chased by cubs	Social Play	Pursued by conspecific
Chase Sirius	Social Play	Runs after conspecific
Chase Shima	Social Play	Runs after conspecific
Chase Serikin	Social Play	Runs after conspecific
Chase Salween	Social Play	Runs after conspecific
Chase cubs	Social Play	Runs after conspecific
Head-butted by Sirius and reciprocates	Social Play	Cat reciprocates interaction with conspecific
Head-butted by Sirius	Social Play	Has its head briefly bumped by a conspecific’s head
Head-butted by Shima and reciprocates	Social Play	Cat reciprocates interaction with conspecific
Head-butted by Shima	Social Play	Has its head briefly bumped by a conspecific’s head
Head-butted by Serikin and reciprocates	Social Play	Cat reciprocates interaction with conspecific
Head-butted by Serikin	Social Play	Has its head briefly bumped by a conspecific’s head
Head-butted by Salween and reciprocates	Social Play	Cat reciprocates interaction with conspecific
Head-butted by Salween	Social Play	Has its head briefly bumped by a conspecific’s head
Head-butted by cubs and reciprocates	Social Play	Cat reciprocates interaction with conspecific
Head-butted by cubs	Social Play	Has its head briefly bumped by a conspecific’s head
Head-butt Sirius	Social Play	Briefly pushes/bumps its head against a conspecific’s head
Head-butt Shima	Social Play	Briefly pushes/bumps its head against a conspecific’s head
Head-butt Serikin	Social Play	Briefly pushes/bumps its head against a conspecific’s head
Head-butt Salween	Social Play	Briefly pushes/bumps its head against a conspecific’s head
Head-butt cubs	Social Play	Briefly pushes/bumps its head against a conspecific’s head
Nape bite Sirius	Social Play	Cat performs an inhibited nape bite, where it will place its mouth on or around the back of a conspecific’s neck but is unlikely to actually bite down
Nape bite Shima	Social Play	Cat performs an inhibited nape bite, where it will place its mouth on or around the back of a conspecific’s neck but is unlikely to actually bite down
Nape bite Serikin	Social Play	Cat performs an inhibited nape bite, where it will place its mouth on or around the back of a conspecific’s neck but is unlikely to actually bite down
Nape bite Salween	Social Play	Cat performs an inhibited nape bite, where it will place its mouth on or around the back of a conspecific’s neck but is unlikely to actually bite down
Nape bite cubs	Social Play	Cat performs an inhibited nape bite, where it will place its mouth on or around the back of a conspecific’s neck but is unlikely to actually bite down
Receives play-fight from Sirius and reciprocates	Social Play	Active receiver of conspecific play
Receives play-fight from Shima and reciprocates	Social Play	Active receiver of conspecific play
Receives play-fight from Serikin and reciprocates	Social Play	Active receiver of conspecific play
Receives play-fight from Salween and reciprocates	Social Play	Active receiver of conspecific play
Receives play-fight from cubs and reciprocates	Social Play	Active receiver of conspecific play
Receive play-fight from Sirius	Social Play	Passive receiver of conspecific play
Receive play-fight from Shima	Social Play	Passive receiver of conspecific play
Receive play-fight from Serikin	Social Play	Passive receiver of conspecific play
Receive play-fight from Salween	Social Play	Passive receiver of conspecific play
Receive play-fight from cubs	Social Play	Passive receiver of conspecific play
Play-fight with Sirius	Social Play	Initiates interaction with conspecific in a non-harmful manner (chasing, jumping, wrestling, etc.)
Play-fight with Shima	Social Play	Initiates interaction with conspecific in a non-harmful manner (chasing, jumping, wrestling, etc.)
Play-fight with Serikin	Social Play	Initiates interaction with conspecific in a non-harmful manner (chasing, jumping, wrestling, etc.)
Play-fight with Salween	Social Play	Initiates interaction with conspecific in a non-harmful manner (chasing, jumping, wrestling, etc.)
Play-fight with cubs	Social Play	Initiates interaction with conspecific in a non-harmful manner (chasing, jumping, wrestling, etc.)
Pawed by Sirius and reciprocates	Social Play	Cat reciprocates interaction with conspecific
Pawed by Sirius	Social Play	Struck by paw of another conspecific
Pawed by Shima and reciprocates	Social Play	Cat reciprocates interaction with conspecific
Pawed by Shima	Social Play	Struck by paw of another conspecific
Pawed by Serikin and reciprocates	Social Play	Cat reciprocates interaction with conspecific
Pawed by Serikin	Social Play	Struck by paw of another conspecific
Pawed by Salween and reciprocates	Social Play	Cat reciprocates interaction with conspecific
Pawed by Salween	Social Play	Struck by paw of another conspecific
Pawed by cubs and reciprocates	Social Play	Cat reciprocates interaction with conspecific
Pawed by cubs	Social Play	Struck by paw of another conspecific
Paw Sirius	Social Play	Strikes someone else with the paw
Paw Shima	Social Play	Strikes someone else with the paw
Paw Serikin	Social Play	Strikes someone else with the paw
Paw Salween	Social Play	Strikes someone else with the paw
Paw cubs	Social Play	Strikes someone else with the paw
Play roll with Sirius	Social Play	Cat rolls onto its back, with its belly exposed and all paws in the air, within a playful context; all agonistic behaviors are absent (i.e., hissing, ears back)
Play roll with Shima	Social Play	Cat rolls onto its back, with its belly exposed and all paws in the air, within a playful context; all agonistic behaviors are absent (i.e., hissing, ears back)
Play roll with Serikin	Social Play	Cat rolls onto its back, with its belly exposed and all paws in the air, within a playful context; all agonistic behaviors are absent (i.e., hissing, ears back)
Play roll with Salween	Social Play	Cat rolls onto its back, with its belly exposed and all paws in the air, within a playful context; all agonistic behaviors are absent (i.e., hissing, ears back)
Play roll with cubs	Social Play	Cat rolls onto its back, with its belly exposed and all paws in the air, within a playful context; all agonistic behaviors are absent (i.e., hissing, ears back)
Steal object from Sirius	Social Play	Steals object from conspecific
Steal object from Shima	Social Play	Steals object from conspecific
Steal object from Serikin	Social Play	Steals object from conspecific
Steal object from Salween	Social Play	Steals object from conspecific
Steal object from cubs	Social Play	Steals object from conspecific
Stalked by Sirius	Social Play	Cat is stalked by conspecific
Stalked by Shima	Social Play	Cat is stalked by conspecific
Stalked by Serikin	Social Play	Cat is stalked by conspecific
Stalked by Salween	Social Play	Cat is stalked by conspecific
Stalked by cubs	Social Play	Cat is stalked by conspecific
Stalk Sirius	Social Play	Usually slow, forward locomotion with back and head slightly lowered, and eyes focused on the stalked individual
Stalk Shima	Social Play	Usually slow, forward locomotion with back and head slightly lowered, and eyes focused on the stalked individual
Stalk Serikin	Social Play	Usually slow, forward locomotion with back and head slightly lowered, and eyes focused on the stalked individual
Stalk Salween	Social Play	Usually slow, forward locomotion with back and head slightly lowered, and eyes focused on the stalked individual
Stalk cubs	Social Play	Usually slow, forward locomotion with back and head slightly lowered, and eyes focused on the stalked individual
Run from Sirius playfully	Social Play	Cat runs away from conspecific
Run from Shima playfully	Social Play	Cat runs away from conspecific
Run from Serikin playfully	Social Play	Cat runs away from conspecific
Run from Salween playfully	Social Play	Cat runs away from conspecific
Run from cubs playfully	Social Play	Cat runs away from conspecific
Object stolen by Sirius and reciprocates	Social Play	Cat reciprocates interaction with conspecific
Object stolen by Sirius	Social Play	Has object stolen by conspecific
Object stolen by Shima and reciprocates	Social Play	Cat reciprocates interaction with conspecific
Object stolen by Shima	Social Play	Has object stolen by conspecific
Object stolen by Serikin and reciprocates	Social Play	Cat reciprocates interaction with conspecific
Object stolen by Serikin	Social Play	Has object stolen by conspecific
Object stolen by Salween and reciprocates	Social Play	Cat reciprocates interaction with conspecific
Object stolen by Salween	Social Play	Has object stolen by conspecific
Object stolen by cubs and reciprocates	Social Play	Cat reciprocates interaction with conspecific
Object stolen by cubs	Social Play	Has object stolen by conspecific
Tripped by Sirius	Social Play	Tripped up by conspecific
Tripped by Shima	Social Play	Tripped up by conspecific
Tripped by Serikin	Social Play	Tripped up by conspecific
Tripped by Salween	Social Play	Tripped up by conspecific
Tripped by cubs	Social Play	Tripped up by conspecific
Trip Sirius	Social Play	Catches the leg of conspecific causing them to fall or stumble
Trip Shima	Social Play	Catches the leg of conspecific causing them to fall or stumble
Trip Serikin	Social Play	Catches the leg of conspecific causing them to fall or stumble
Trip Salween	Social Play	Catches the leg of conspecific causing them to fall or stumble
Trip cubs	Social Play	Catches the leg of conspecific causing them to fall or stumble
Jumped on by Sirius playfully	Social Play	Attacked suddenly and playfully jumped on by conspecific
Jumped on by Salween playfully	Social Play	Attacked suddenly and playfully jumped on by conspecific
Jumped on by Shima playfully	Social Play	Attacked suddenly and playfully jumped on by conspecific
Jumped on by Serikin playfully	Social Play	Attacked suddenly and playfully jumped on by conspecific
Jumped on by cubs playfully	Social Play	Attacked suddenly and playfully jumped on by conspecific
Jump on Sirius playfully	Social Play	Attack suddenly and playfully jump on the back of conspecific
Jump on Salween playfully	Social Play	Attack suddenly and playfully jump on the back of conspecific
Jump on Shima playfully	Social Play	Attack suddenly and playfully jump on the back of conspecific
Jump on Serikin playfully	Social Play	Attack suddenly and playfully jump on the back of conspecific
Jump on cubs playfully	Social Play	Attack suddenly and playfully jump on the back of conspecific
Play with water	Solitary Play	Cat interacts with something in a non-serious manner (i.e., where there is no intention to harm)
Play with object	Solitary Play	Cat interacts with something in a non-serious manner (i.e., where there is no intention to harm)
Play roll	Solitary Play	Cat rolls onto its back, with its belly exposed and all paws in the air, within a playful context; all agonistic behaviors are absent (i.e., hissing, ears back)
Carry object	Solitary Play	Cat carries an object in its mouth
Pace	Stereotypic	Repetitive locomotion in a fixed pattern
Body rub object	Territorial	Rubs body on object
Head rubbing object	Territorial	Cat rubs its head against an object
Spray	Territorial	Stands with tail raised vertically and releases a jet of urine backwards against a vertical surface or object
Scratching with paws	Territorial	Cat scratches an object using the claws of its fore feet
Vocalization—Syndetic call	Vocalization	Amiable call with the purpose of gather or appease conspecifics
Vocalization—Roar	Vocalization	Long, throaty, high intensity call
Vocalization—Hiss	Vocalization	A drawn-out, low-intensity hissing sound produced by rapid expulsion of air from the cat’s mouth, usually during exhalation
Vocalization—Grunt/Cough	Vocalization	Short, throaty call, characterized by the deep contraction and expansion of the diaphragm
Vocalization—Growl	Vocalization	A low-pitched, throaty, rumbling noise produced while the mouth is closed
Vocalization—Chuff	Vocalization	Cat expels jets of air through the nose creating a low intensity, soft, pulsed sound, described as being similar to the snorting of a horse
